# COVID-19 Pandemic Impact and Response in Canadian Pediatric Chronic Pain Care: A National Survey of Medical Directors and Pain Professionals

**DOI:** 10.1080/24740527.2021.1931069

**Published:** 2021-06-30

**Authors:** Tieghan Killackey, Melanie Noel, Kathryn A. Birnie, Manon Choinière, M. Gabrielle Pagé, Lise Dassieu, Anaïs Lacasse, Chitra Lalloo, Sarah Brennenstuhl, Patricia Poulin, Pablo Ingelmo, Samina Ali, Marco Battaglia, Fiona Campbell, Andrew Smith, Lauren Harris, Vina Mohabir, Myles Benayon, Isabel Jordan, Justina Marianayagam, Jennifer Stinson

**Affiliations:** a The Hospital for Sick Children, Child Health Evaluative Sciences, Toronto, Ontario, Canada; b Alberta Children’s Hospital, Hotchkiss Brain Institute, University of Calgary, Calgary, Alberta, Canada; c Department of Anesthesiology, Perioperative, and Pain Medicine, Alberta Children’s Hospital Research Institute, University of Calgary, Calgary, Alberta, Canada; d Department of Anesthesiology and Pain Medicine, Faculty of Medicine, Université de Montréal, Montreal, Quebec, Canada; e Research Center of the Centre Hospitalier de l’Université de Montréal, Montreal, Quebec, Canada; f Department of Anesthesiology and Pain Medicine, Faculty of Medicine, Department of Psychology, Université de Montréal, Montreal, Quebec, Canada; g Department of Biomedical Sciences, Université de Montréal, Montreal, Quebec, Canada; h Department of Health Sciences, Université du Québec en Abitibi-Témiscamingue, Rouyn-Noranda, Quebec, Canada; i Institute of Health Policy, Management & Evaluation, University of Toronto, Toronto, Ontario, Canada; j Lawrence S. Bloomberg Faculty of Nursing, University of Toronto, Toronto, Ontario, Canada; k Department of Anesthesiology & Pain Medicine, University of Ottawa, Ottawa, Ontario, Canada; l Department of Psychology, The Ottawa Hospital, Ottawa, Ontario, Canada; m Clinical Epidemiology Program, The Ottawa Hospital Research Institute, Ottawa, Ontario, Canada; n Chronic Pain Service, Montreal Children’s Hospital, McGill University Health Center, Montreal, Quebec, Canada; o Departments of Pediatrics & Emergency Medicine, Faculty of Medicine & Dentistry, University of Alberta, Edmonton, Alberta, Canada; p Women and Children’s Health Research Institute, University of Alberta, Edmonton, Alberta, Canada; q Department of Psychiatry, University of Toronto, Toronto, Ontario, Canada; r Division of Child Youth & Emerging Adulthood Psychiatry, CAMH, Toronto, Ontario, Canada; s Department of Anesthesia and Pain Medicine, Hospital for Sick Children, University of Toronto, Toronto, Ontario, Canada; t Interprofessional Pain and Addiction Recovery Clinic, Centre for Addiction and Mental Health, Toronto, Ontario, Canada; u Department of Medicine, McMaster University, Hamilton, Ontario, Canada; v British Columbia, Canada; w Northern Ontario School of Medicine, Thunder Bay, Ontario, Canada

**Keywords:** COVID-19, pediatric pain, pain clinics, distance treatments, e-health, telehealth

## Abstract

**Background**: The COVID-19 pandemic presents one of the greatest threats to pediatric pain care seen in generations. Due to public health restrictions, many pediatric pain clinics halted in-person appointments, delaying and disrupting access to care. There is no existing research on the impacts of COVID-19 on pediatric chronic pain care in Canada or the challenges experienced by health care professionals and pain clinics.

**Aims**: The aim of this study was to evaluate the impact of COVID-19 on Canadian pediatric chronic pain care by documenting how health care professionals provided care during the first six months of the pandemic.

**Methods**: Two Canadian online cross-sectional surveys were conducted: one among Canadian pediatric pain clinic directors (Study 1) and another among multidisciplinary pediatric pain health care professionals (Study 2).

**Results**: Responses from 13/13 Canadian pediatric pain clinics/rehabilitation programs indicated that all clinics provided virtual care during the pandemic. No significant changes were reported on the frequency of appointment requests. Most clinics reported no perceived change in patient pain levels (*n* = 9/13, 69%) or occurrence of pain flares (*n* = 10/13, 77%). Results from 151 individual health care professionals indicated that the majority (90%) of non–emergency department respondents were providing virtual care. The main challenges of virtual care included technological barriers, financial concerns, infrastructure and logistics, privacy, and clinical challenges.

**Conclusions**: This study documented the impact of the COVID-19 pandemic on pediatric chronic pain care in Canada and highlighted the rapid shift to using virtual solutions. Simultaneously, respondents outlined current challenges and potential solutions to consider in the development of virtual care guidelines and policy in Canada.

## RÉSUMÉ

**Contexte:** La pandémie de COVID-19 représente l’une des plus grandes menaces pour les soins de la douleur pédiatrique jamais connue depuis des générations. En raison de restrictions de santé publique, de nombreuses cliniques pédiatriques de la douleur ont interrompu les rendez-vous en personne, occasionnant ainsi des retards et des perturbations dans l’accès aux soins. Il n’existe pas de recherche sur les répercussions de la COVID-19 sur les soins pédiatriques de la douleur chronique au Canada ou sur les défis rencontrés par les professionnels des soins de santé et les cliniques de la douleur.

**Objectifs:** L’objectif de cette étude était d’évaluer les répercussions de la COVID-19 sur les soins pédiatriques de la douleur chronique au Canada en documentant la façon dont les professionnels de la santé ont prodigué les soins au cours des six premiers mois de la pandémie.

**Méthodes:** Deux enquêtes transversales canadiennes en ligne ont été menées : l’une auprès des directeurs de cliniques pédiatriques de la douleur canadiennes (Étude 1) et l’autre auprès de professionnels des soins de santé de la douleur pédiatriques multidisciplinaires (étude 2).

**Résultats:** Les réponses obtenues de la part de 13 / 13 cliniques pédiatriques de la douleur / programmes de réadaptation ont indiqué que toutes les cliniques avaient fourni des soins virtuels pendant la pandémie. Aucun changement significatif n’a été rapporté sur la fréquence des demandes de rendez-vous. La plupart des cliniques n’ont signalé aucun changement perçu dans les niveaux de douleur des patients (n = 9/13, 69 %) ou la survenue de poussées douloureuses (n = 10/13, 77 %). Les résultats obtenus de 151 professionnels de la santé ont indiqué que la majorité (90 %) des répondants de département autres que les urgences fournissaient des soins virtuels. Les principaux défis des soins virtuels comprenaient les barrières technologiques, les préoccupations financières, l’infrastructure et la logistique, la confidentialité et les défis cliniques.

**Conclusions:** Cette étude a documenté les répercussions de la pandémie de COVID-19 sur les soins pédiatriques de la douleur chronique au Canada et a mis en lumière le passage rapide à l’utilisation de solutions virtuelles. Simultanément, les répondants ont décrit les défis actuels et les solutions potentielles devant être tenues en compte dans l’élaboration de lignes directrices et de politiques sur les soins virtuels au Canada.

## Introduction

Chronic pain affects 20% of Canadian youth^1^ and can negatively impact all aspects of life and development. Many youth with chronic pain are socially isolated and have co-occurring mental health issues.^2,3^ Childhood chronic pain costs US$19B annually in direct and indirect costs in the United States, exceeding expenditures for childhood obesity and asthma.^4–6^ Early pain intervention can reduce pain duration, disability, and health care costs. The optimal treatment model for pediatric chronic pain is a biopsychosocial “3-P” approach that involves pharmacological, physical, and psychological strategies.^7^ In general, the most severe and highly impaired individuals are seen in tertiary-level clinics, and the majority are managed by community professionals.^8,9^

Poor access to care for youth living with chronic pain has been identified as a longstanding problem and research priority for patients and families.^7,10^ If health care services or care delivery is compromised, youth with pain are at risk of developing mental health concerns (including posttraumatic stress disorder [PTSD], depression, anxiety, and suicide), deteriorations in functioning, and substance use disorder.^11,12^ Moreover, many youth living with pain have previously existing mental health comorbidities, which places them at even greater risk.^13^ Starting in mid-March 2020 when widespread public health restrictions began in Canada, young people with chronic pain and their families have faced many challenges as ambulatory chronic pain programs halted new in-person consults, follow-up appointments, and individual therapy sessions. Moreover, many community-based therapists (e.g., physiotherapists [PTs], psychologists) and clinics halted in-person appointments^14^ and school closures also suddenly prevented access to services such as counselors and social workers.

Emerging pain research has focused on characterizing the impacts of COVID-19 on health care delivery^15^ and psychosocial variables^16^ as well as considerations for implementing eHealth services.^17^ However, this research has been primarily adult focused and does not take into account the unique needs of youth living with pain or how pain intersects with other key developmental milestones in adolescence. In order to rapidly deploy feasible solutions for this highly vulnerable population during and beyond the pandemic, it is critical to understand the experience of medical directors and health care professionals who support youth living with chronic pain.

The overarching aims of this study were to evaluate the impact and experiences of the COVID-19 pandemic on (1) Canadian pediatric pain clinics and (2) health care professionals working in the pediatric chronic pain context. This work builds on and extends research done in the adult chronic pain setting,^15,18,19^ specifically Lynch et al.,^15^ who documented the impact of COVID-19 and response through a national survey of adult pain clinics. This study is part of a larger, multiphase project supported by a rapid grant from the Canadian Institutes for Health Research with a focus on mental health and substance use service needs and delivery. This broader project seeks to understand impacts of the COVID-19 pandemic on the pain experience, mental health, and substance use among youth with chronic pain, their siblings, and parents; this component of the project sought to obtain a rapid understanding of how health care professionals (HCPs) are providing pediatric chronic pain care during the pandemic.

## Methods

### Design

Two online cross-sectional surveys were designed to explore the impact and experiences of Canadian pediatric pain clinics and pediatric pain care professionals in the context of the COVID-19 pandemic. The surveys included questions about the impact on services that were previously offered, impacts on patients, obstacles encountered with virtual care, and whether new initiatives would continue after the pandemic. The Study 1 survey was a modified version of a similar survey administered to adult pain clinic directors.^15^ In addition to those published in full, questions were added to assess any changes specific to the mental health needs at each clinic (i.e., “Have you seen an increase of [anxiety/posttraumatic stress/depressive] symptoms?”) as well as questions regarding screening for substance use disorders, in order to attend to the specific focus of the funding call on mental health and substance use during the pandemic.

The Study 2 survey was modified specifically for health care professionals delivering pain care in the community and in emergency departments, with input from key clinicians in each of those areas to ensure the questions were relevant and appropriate. All surveys were tested by at least three target end-users (i.e., clinical nurses, psychologists, etc.) for usability, ease of completion, and time to complete prior to data collection. The surveys were approved by the Hospital for Sick Children Research Ethics Board (#1000070100) and electronic informed consent was obtained from participants completing the survey; both surveys can be found in the supplementary material.

### Study 1

The Study 1 survey was distributed by e-mail to all medical directors of multi-/interdisciplinary pain clinics and rehabilitation programs across Canada (*n* = 13). Medical directors were chosen to be surveyed in order to obtain insight into institutional or organizational level changes that accompanied the COVID-19 pandemic and report on the state of tertiary pediatric chronic pain care provision across Canada; this approach was also based on a similar survey conducted with medical directors of Canadian adult pain clincs and we followed this precedent in order to collect comparable data.^15^ Medical directors (or a delegate) were selected specifically in order to gather a pan-Canadian perspective, while minimizing duplication of responses regarding (1) impact on each chronic pain clinic and its patients and (2) organizational-level enablers and barriers to care. Clinic directors were asked to complete the survey themselves or forward to a delegate who would be able to answer questions about the changing current care delivery models of the clinic as well as the impact of the pandemic on clinic patients.

### Study 2

The Study 2 survey was distributed using a convenience sampling approach, targeting a number of networks where patients with pain would be likely to present: primary care professional networks via the College of Family Physicians of Canada Member Interest Group (Montgomery), chronic pain professionals via the Pediatric Pain Network and the Canadian Pain Task Force (Campbell), and pediatric rheumatologists via the Canadian Alliance of Pediatric Rheumatology Investigators (Stinson), and emergency department physicians affiliated with Pediatric Emergency Research Canada (Ali). Clinic directors and other respondents were asked to fan the survey out among their pediatric pain networks for maximum reach. Individuals were excluded if they did not provide clinical care to the pediatric chronic pain population. A secure web application (REDCap) was used for building, collecting, and managing the survey data. Participants were asked to complete the survey within two weeks, and one or two reminder e-mails were sent after a week to increase responses. Surveys were available in English and French, and data were collected between June and September 2020; in Canada, this was following the first wave of the pandemic and respondents were being asked about their experience during the intensive restrictions and closures that occurred during the spring. Data were de-identified and aggregated.

### Data Analysis

The demographic characteristics of samples and quantitative survey data were analyzed using frequency counts and percentages. Because of the differences in care provided in community-based and emergency department (ED) settings, these groups were disaggregated. When comparisons were made, chi-square test or Fisher’s exact test (when expected cell counts were below five) was used. Data from open-ended survey questions were analyzed by the lead author using content analysis^20^ to categorize data; key categories were developed by organizing the data into broad themes.^20^

## Results

### Study 1: COVID-19 Pandemic Impact on Pain Clinics

#### Impact on Pain Clinic Services

All pediatric chronic pain clinic directors in Canada (*N* = 13/13) responded to the survey. The clinics provide an average of 751 patient visits per year (range 25–2613) and combined have a total of 8268 patient visits per year. Survey results from the medical directors are displayed in Table 1. The vast majority (*n* = 12/13, 92%) reported not having received an increase in requests for opioid or other analgesic prescriptions or seeing an increase in substance use disorders (*n* = 12/13, 92%). The majority (85%, *n* = 11/13) reported that clinics were triaging new patients and deciding to either see using virtual care or add to a waiting list.

**Table 1.** Pain clinic survey results

Service delivery	Responses, *N* (%)
Has your clinic had to stop or significantly reduce in-person patient appointments?	Yes: *n* = 12 (92)
	No: *n* = 1 (8)
Are you providing care by other means?	
Internet/video calling	*N* = 13 (100)
Telephone	*N* = 10 (77)
Primary obstacles for virtual care delivery	
Technological issues (i.e., Internet connection, access to technology, technological literacy)	*N* = 7 (54)
Administrative and logistical barriers related to infrastructure and setting up virtual care capabilities	*N* = 6 (46)
Are you still delivering physical, psychological, and/or educational programs, either in person or virtually?	Yes: *n* = 13 (100)
	No: *n* = 0 (0)
Number of appointment requests	
Have you seen an increase or decrease in the number of requests to be seen from existing chronic pain patients?	Increase: *n* = 2 (15) No change: *n* = 7 (54) Decrease: *n* = 4 (31)
Have you had an increase or decrease in consults for new chronic pain patients?	Increase: *n* = 3 (23)
	No change: *n* = 7 (54)
	Decrease: *n* = 3 (23)

All medical directors (*N* = 13/13, 100%) reported routinely screening patients for anxiety, depression, and insomnia or sleep disturbances. Only half reported routinely screening for PTSD (*n* = 6/13, 46%) or substance use disorders (*n* = 7/13, 54%). The types of screening methods used varied, where six clinics (46%) reported using standardized questionnaires, five clinics (38%) reported using clinical interview methods, and two clinics (15%) reported using a combination of both. Most clinic directors reported that they did not have a formal pathway to refer youth with chronic pain to substance use disorder counselling services (*n* = 10/13, 77%). Clinic directors also reported a number of barriers to access for both mental health and substance use disorder services directly related to COVID-19, including significant waitlists (*n* = 8/13), lack of available services in the community (*n* = 7/13; especially challenging in certain geographic areas), financial constraints of patients (*n* = 3/13), and the need for mental health concerns to be especially severe (i.e., suicidal ideation) in order to qualify for treatment (*n* = 2/13).

#### Impact on Pain Clinic Patients

Most pediatric chronic pain clinic directors reported that from their perspectives, patients were not reporting increases in pain intensity (*n* = 9/13, 69%), number of chronic pain flares (*n* = 10/13, 77%), or flares leading to hospital admission (*n* = 11/13, 85%). Furthermore, there was no perception that patients were increasing medication use (*n* = 8/13, 62%), and the majority did not note patient issues related to medication (i.e., prescribing, procuring, etc.) during the pandemic (*n* = 11/13, 85%). However, directors who did report observing an increase in medication use (*n* = 5/13) reported observing increases primarily of adjuvant pain medications including antidepressants and anticonvulsants. The majority of chronic pain clinics reported no perceived changes in their patients exhibiting mental health symptoms such as PTSD (**n** = 12/13, 92%) and insomnia (*n* = 9/12, 75%); however, some clinic directors did perceive increases in both anxiety (*n* = 5/13, 38%, in Alberta, Saskatchewan, Quebec, and Ontario) and depressive (*n* = 6/13, 46%, in Alberta, Ontario, and Quebec) symptoms.

### Study 2: COVID-19 Pandemic Impact on Pediatric Health Care Professionals

#### Participants’ Demographics

Study 2 consisted of a survey of Canadian health care professionals who treated pediatric chronic pain across the continuum of care, including pediatric pain clinic and primary care professionals (*n* = 103) and pediatric ED professionals (*n* = 48), for a total sample of 151 health care providers. Overall, three-quarters of participants were female (75%; *n* = 113); significantly more females respondents were from community settings (81.4%) compared to ED settings (62.5%; *P* = 0.03). The largest share of respondents came from Ontario (40%; *n* = 60), with a significantly higher frequency than those in community settings residing in Ontario (51.0%) compared to ED settings (10%; *P* = 0.001). Table 2 includes a breakdown of the clinical roles of study participants. Participants who did not work in the ED setting primarily worked in a pediatric hospital (74%; *n* = 76) or tertiary pain clinic (24%; *n* = 25) and, overall, participants reported a range of experience working with youth with chronic pain, from less than one year to over 15 years.

**Table 2.** Breakdown of roles of survey respondents

Health care provider role	*N* (%)
Physician	86 (57)
Anesthesiologist	13 (9)
Pediatrician	16 (11)
Family physician	3 (2)
Psychiatrist	3 (2)
Pediatric emergency physician	43 (28)
Other	8 (5)
Nurse	17 (11)
Registered nurse	5 (3)
Advanced practice nurse	7 (5)
Nurse practitioner	5 (3)
Psychologist	17 (11)
Physical therapist	17 (11)
Occupational therapist	3 (2)
Social worker	4 (3)
Pharmacist	2 (1)
Other	5 (3)
Total	151 (100)

#### Impact on Pediatric Health Care Professionals

Table 3 outlines responses to survey items related to impact of care by setting type. More than 90% (*n* = 94/103) of non-ED professionals reported providing care by means other than in-person visits, and this did not vary between the two hardest hit regions of Ontario and Quebec and elsewhere (89.1% vs. 94.9%; *P* = 0.31). The vast majority (85%) also reported having to halt or significantly reduce in-person appointments due to the COVID-19 pandemic. Non-ED participants reported primarily using Internet video calling (86.4%) and telephone calls (67.0%), and the largest share used Zoom for Healthcare^21^ (58.3%), followed by provincial systems (i.e., Ontario Telemedicine Network; MBTelehealth; 47.6%) to conduct appointments. Overall, the majority (61%) of non-ED professionals reported that they were still providing physical, psychological, or educational programs (either in person or virtually), and since implementing virtual care, 46% (*n* = 47/103) reported improved appointment attendance. The vast majority (85%; *n* = 41/48) of participants working in the ED setting reported that they were not providing virtual care, and though this proportion was slightly lower in the two hardest hit regions of Ontario and Quebec compared to the rest of the provinces represented in the study, the difference was not significant (82.1% vs. 94.7%; *P* = 0.20). Though this survey focused on the impact on care delivery for patients living with chronic pain, one finding related to the volume of ED patients presenting during the current pandemic. Of the ED respondents, 90% (*n* = 43/48) noted that there was a decrease in daily volume of patients; however, those patients who did attend the ED were reported to have higher levels of illness severity and acuity.

**Table 3.** Responses to survey items related to impact of care by setting type

Impact	Non-ED settings (*n* = 103), *n* (%)	ED (*n* = 48), *n* (%)
Impact on service delivery		
Had to stop or significantly reduce in-person appointments	88 (85.4)	n/a
Providing care other than in person	94 (91.3)	7 (14.6)
Use Internet/video calling	89 (86.4)	5 (10.4)
Use telephone without video	69 (67.0)	4 (8.3)
Use other form of care not in person (e.g., e-mail)	3 (2.9)	1 (2.1)
Use Zoom for video calls	60 (58.3)	3 (6.3)
Use provincial system for video calls	49 (47.6)	0 (0.0)
Use other program for video calls	16 (15.5)	2 (4.2)
Still providing physical, psychological, or educational programs (either in person or virtually)	63 (61.2)	n/a
Change in rate of attendance		
No change	26 (25.2)	n/a
Yes, improved attendance	47 (45.6)	n/a
Yes, worse attendance	15 (14.6)	n/a
Not applicable	15 (14.6)	n/a

ED = emergency department.

#### Challenges and Opportunities with Virtual Care

Responses to open-ended questions revealed a variety of challenges professionals encountered with the rapid introduction of virtual care delivery models that impacted their ability to provide care (see Table 4), along with identifying many benefits and future opportunities related to virtual care (see Table 5).

**Table 4.** Reported challenges of delivering care virtually

Type of challenge	Examples of challenge	Open-ended survey: Participant quotes
Technological	Connection issues (from both patient and HCP end) Patients having limited technological literacy (i.e., navigating virtual care platforms) HCPs and patients having limited technical support and resources	“Patient and family appropriate video capability and bandwidth” “Issues with technical literacy (patient not sure how to navigate mic/camera settings)” “Families who live in rural settings often have poor Internet connections” “Technical difficulties with home based worked and VPN and global protect” “Do not have the actual equipment or IT support”
Financial	Limited access to technology needed to conduct virtual care Limited access to affordable and reliable Internet Billing concerns	“Not all patients have access to reliable Wi-Fi, smartphones/laptops, etc.” “Family does not have laptop with headphones, no video, etc.” “Poor patient Internet access”
Infrastructure or logistical	Selecting platform Physical space requirements (at home and at work) Securing administrative support Triaging care	“Not everyone on my team has an OTN or Zoom health care account, making access difficult” “Wi-Fi does not work in all parts of hospital” “Lack of equipment and space” “Figuring out logistics; e.g., what platform would be used to see patients and document visit, how patients would access visit, what kind of physical space would be needed, what types of patients would be seen, how would patients access the services, etc.” “It took us some time to get our user agreement set up and appointment templates, etc.” “Lack of admin support”
Privacy concerns	Platform security (i.e., concerns that patients could login to other patients’ sessions) Confidentiality of virtual appointments (i.e., have to take calls at home where parents or family members could be listening in on confidential information)	“OTN locked but have had one case another patient entered another patients visit” “Availability of safe connections, stability of system” “No easy, secure video platform made available” “Harder to meet with kids and teens independently—private spots often have poorer Wi-Fi, and families are usually at home during pandemic, so ensuring confidentiality is more challenging” “Patient/parent access to confidential safe space”
Clinical	Limited ability to conduct physical assessments and hands-on examinations Limited ability to assess psychological or social–emotional factors (i.e., body language) Challenges building rapport through a virtual interface (vs. in person)	“I can’t physically examine the patients, which is fine for patients I’ve been following but makes me feel less comfortable for new consults. I also can’t do procedures” “Unable to complete physical exam, which a key part of a PT assessment” “Can be hard to do interdisciplinary care virtually” “Inability to adequately view the body, inadequate physicals, no ability to do hands-on” “A lot of clinical time is used trouble-shooting technology (sound, picture cutting out, dropped calls, etc.)” “Assessment of psychological and social–emotional factors via video is much more challenging and artificial” “Therapeutic rapport building”

HCP = health care professional; VPN = virtual private network; IT = information technology; OTN = Ontario Telemedicine Network; PT = physical therapy.

**Table 5.** Identified opportunities and solutions to reported challenges related to virtual care delivery

Type of challenge	Examples of opportunities and solutions	Open-ended survey: Participant quotes
Technological	Federal investment in Internet infrastructure to improve access and affordability Programs to enhance technological literacy and provide IT support	“Infrastructure investment to ensure adequate service is available, even in remote and rural areas” “Sometimes the family needs to be educated prior to the appointment. It would help if someone could go through the steps and technical requirements prior to the appointment” “Hospitals and schools should be providing families with IT support”
Financial	Federal investment in Internet infrastructure to improve access and affordability Government grants for low-income families to purchase technology Programs to loan hardware to families in need Improved billing codes and systems for virtual care	“Greater funding to support marginalized families to have access to technology” “If children and youth will be relying on Wi-Fi for both education and health care (which fall under the province’s responsibility), more needs to be done to ensure highiquality, affordable Wi-Fi is more readily available to all areas (including remote areas). Grants may be needed to provide families with rebates for investing in technology to support their children’s health and educational needs. Stricter regulations on for-profit Wi-Fi providers are needed to ensure families have affordable access to both quality Wi-Fi and data plans, since data is often needed to substitute for poor Wi-Fi” “Improved Internet resources and accessibility for those with low socioeconomic status would improve their access to care” “Provide free access for patient and families for hardware and software” “The province could potentially strengthen networks with infrastructure in more remote areas of the province. There could be an additional program through government assistance programs (like ODSB) that provides access to adequate Internet to support virtual care. Much like they did for students who needed assistance accessing online learning during COVID” “Internet and wireless needs to be viewed as an essential utility” “Families being given technology or loaned it, in the absence of technology, or a grant for families to access funding to purchase something so that they can continue their care during a pandemic” “Ongoing support for physicians to bill for virtual care at the same level as in person care” “In general, there needs to be billing codes that cover virtual care”
Infrastructure or logistical	Development of guidelines and best practices for pediatric virtual care Tailored education and development of competencies to support professional training Increased IT support Funding available for platform licenses	“Appropriate IT support” “Support for staff around using virtual platforms (training)” “Someone/a team with dedicated time and motivation to figure out logistics” “Better office support to organize logistics of appointments” “Help with a platform, as we do not have one in the emergency setting” “Providing more secured licenses for virtual practice” “Funding for equipment, paid training for offering virtual care”
Privacy concerns	Creating a software that meets security requirements Local virtual care centers (i.e., similar to current telehealth centers, patients can attend a local center and use computer and Internet services to participate in virtual care)	“Creating an easy software platform that meets provincial security requirements” “Listing of virtual care centers (or increase number) for families to go to locally if unable to do virtual visit at home with own equipment” “New OTN centers patient/families can go to that include safe space, and confidential space” “Ensure access to technology hubs in hard to reach areas may also help bridge the gap of patients with chronic pain which we are not seeing referred to our clinic” “Funding for infrastructure and increased staffing”
Clinical	Hybrid model of care delivery (combined virtual and face-to-face) Virtual stepped care models (i.e., virtual models that match patients with the individualized care that they need)	“Will bring in person as needed or will do physical assessment over zoom as best we can” “Trial of virtual care and studying the outcomes of the patient (preventing ED visits, creating double virtual care and ED visit, missing sick patients)” “Integrated stepped care program—move them up to get right care at right time by right person secure technology solutions”

IT = information technology; ODSB = Ontario Disability Support Program; OTN = Ontario Telemedicine Network; ED = emergency department.

The most significant barrier to virtual care provision reported by non-ED professionals was technological (65%). These concerns related primarily to (1) connection issues such as bandwidth, Internet speeds, call freezing or virtual private network challenges; (2) lack of access to technology or limited technological literacy (i.e., no video capability, sound problems, uncomfortable using platforms); (3) challenges using platforms (i.e., link does not work, patient stuck in electronic waiting room on digital platform and no one is notified); and (4) limited support or resources (i.e., limited licenses for platforms, limited local information technology [IT] support). Technological challenges were often closely linked to financial challenges experienced primarily by patients and families but also by health care professionals and institutions. Participants reported that many families did not appear to have access to the technology and struggled with securing reliable Internet connections needed to participate in virtual care. Some HCPs also reported that their institutions were unable to purchase the amount of equipment needed for virtual care and also expressed their concerns about being able to adequately bill for virtual services.

Some professionals described several benefits with the use of virtual care, including convenience of conducting follow-up visits not requiring a physical examination, meeting with patients who live in rural or remote locations, and increased accessibility for those who face financial obstacles traveling to hospital. Many professionals had begun or planned to initiate a hybrid model, whereby either (1) some clinicians are onsite and see patients in-person while others work virtually or (2) some clinic days are reserved specifically for virtual care with patients and others are for in-person visits.

In order to address the previously outlined technological and financial concerns, professionals described a need for investment in improved Internet infrastructure across the country, especially in rural and remote areas; some expressed that the Internet should be viewed as an essential service and that government assistance should be provided to ensure that everyone has equitable and reliable access. Some participants observed economic challenges experienced by some families and suggested financial support-based strategies such as loaning technology to families or providing grants to ensure that families could purchase the technology needed for virtual health care. Participants also suggested increasing the number of virtual care centers where families can attend locally if unable to complete virtual visits in home (i.e., due to Internet, technology, or privacy concerns). Beyond this, professionals reported that they require more technology to conduct virtual care, increased IT support, training for staff and patients, and funding for platform licenses. Pain health care professionals suggested an integrated stepped care platform^22,23^ that engages users in a range of established and emerging online programs systematically. In this model, the intensity of the program can be either stepped up or down depending on patients’ needs. Finally, professionals reported a need for guidelines and standards of care that could guide the implementation of virtual care and increased education and competencies. Overall, though participants who used virtual care reported benefits for professionals, patients, and patients’ families, they also described the need to ensure equitable access to virtual care despite geography, financial status, or other barriers (see Table 4).

Beyond technological and financial issues, participants reported a number of general challenges with using virtual care. When initially establishing virtual care practices, participants reported challenges making decisions regarding infrastructure and logistics and platform selection (if not predetermined by institution) and training, determining physical space requirements and determining which patients could be seen virtually (i.e., follow-up vs. new appointments), and deciding how patients would access the services. Participants reported significant concerns related to privacy (i.e., patient accidentally entering another patient’s virtual session) and confidentiality, which was especially relevant for teens, who may prefer to meet independently without parents or family overhearing the visit. Professionals expressed that they were challenged or unable to complete appropriate physical assessments and hands-on physical examinations, which was of specific concern for new consultations and certain populations such as children with disabilities or the very young. Participants also had trouble assessing psychological and social–emotional factors via video, leading to perceived challenges building therapeutic rapport. Finally, many participants reported a lack of required resources such as administrative support, IT equipment, and physical space to conduct virtual visits.

## Discussion

The aim of this study was to document how the COVID-19 pandemic has impacted the delivery of care for youth living with chronic pain from the perspective of health care professionals in Canada. A key finding of this work was that pandemic-related restrictions to in-person visits resulted in these therapies being delivered virtually at all 13 of the pediatric pain clinic sites; notably, clinic directors perceived increases in both anxiety and depressive symptoms among patients and highlighted the major challenges that currently exist in ensuring sufficient access to mental health and substance use disorder services, which were already of concern long before the pandemic. Mental health symptoms (such as depression, anxiety, PTSD, insomnia) have been found to be elevated in youth with chronic pain,^13^ and youth with chronic pain are at heightened risk for developing mental health comorbidities over time. The stress and isolation of the COVID-19 pandemic may exacerbate this risk, and comprehensive assessment of youth with chronic pain now routinely includes assessment of aspects of mental health (e.g., in the national pediatric pain registry, outcomes in clinical trials, etc.), especially because increases in mental health symptoms can lead to a worsening of pain over time.^13,24^ Many youth with chronic pain have co-occurring mental health issues,^2,3^ which are routinely screened for at tertiary care pain clinics, yet our results indicate that only half of the clinics currently screen for PTSD and substance use disorder. Additionally, in Canadian adolescents, a frequent pain trajectory predicted fourfold greater odds of receiving a first opioid prescription by age 19, compared to a “none-to-minimal” pain trajectory,^25^ and in the United States, adolescents with chronic pain are at increased risk for future opioid misuse, even adjusting for opioid prescription.^26^ Moreover, more than 50% of adolescent opioid misusers report misuse to self-treat pain, whereas fewer misuse to get “high.”^27^ These data indicate the importance of screening for mental health concerns and substance use disorder in the pediatric pain clinic setting, which were key aspects of this study. There is evidence to suggest that many pediatric clinics and health care providers are missing out on identifying youth who are at high risk for substance use disorder (especially during crisis), and during the COVID-19 pandemic Canada has witnessed steady growths in opioid-related harms, overdoses, and deaths.^28^ Compared to pre-COVID figures, the numbers of admissions per day for substance-related issues appear especially pronounced for the younger strata of the population and may be underestimated, because available rates of substance-related morbidity and mortality are reported only for the hospital settings.^29^ Therefore, there is a need to develop consensus on what screening tools to use in both tertiary and community care outpatient settings and how best to implement these tools in clinical practice. As noted by a recent report by the Royal Society of Canada, mental health care in Canada was already overextended and underresourced, and the COVID-19 pandemic has exacerbated these significant and long-standing weaknesses in the mental health system.^30^

The implementation of virtual care relies on the availability of accessible and functional technology as well as a stable Internet connection, because variable Internet capacity can interrupt appointments and thereby affect the communication. The second report of the Canadian Pain Task Force identified the limited or complete lack of Internet in some rural and remote communities as a barrier to accessing chronic pain care in Canada, a point clearly echoed by our participants.^31^ Participants were also concerned that the use of virtual care could contribute to growing inequities in health care access across Canada, and many respondents in this survey suggested that broad government initiatives, such as greater investment in Internet infrastructure and rapid provision of grants to low-income families, are needed to address these concerns.

Beyond Internet and technological issues, survey respondents noted challenges with infrastructure and logistics. These were also likely functions of having pivoted so quickly to virtual care,^32^ which may result in gaps in education and training for health care professionals and patients, limited resources to guide practice (i.e., practice standards), and challenges in obtaining funding for technology and licenses. Decisions such as selecting a virtual care platform (if given the choice) can be challenging, and health care professionals face many conflicting considerations such as the purpose of the platform,^33,34^ audio/video quality,^17^ and the ability of patients to engage with the platform. A recent topical review further outlined similar challenges with implementing telehealth multidisciplinary pain care in the context of COVID-19 and suggested an agenda of research, education, and policy recommendations to address many of the challenges highlighted in this study, including access, equity, safety, and security. Privacy concerns were also noted as a challenge in this study, which is an especially relevant consideration when providing care to youth and young adults.

Finally, the rapid implementation of virtual care led to new clinical challenges for health care professionals, specifically through the limited ability to conduct complete physical assessments, difficulties assessing social and emotional factors, and the overall struggle to build rapport with the patient and family without in-person interactions. A potential solution to these challenges is a hybrid model of care that allows for a mix of in-person and virtual visits, which has been supported by literature in the pediatric field.^35,36^ In this study, many professionals had begun or planned to initiate a hybrid model after experiencing the benefits of virtual care, whereby either (1) some clinicians are onsite and see patients in person while others work virtually or (2) some clinic days are reserved specifically for virtual care with patients while others are for in-person visits. The Canadian Pain Task Force report from October 2020^31^ reflects on the impact of the COVID-19 pandemic by highlighting the opportunities for implementing innovative patient-centered care models, such as the Stepped Care Model.^22^ Stepped care is a resiliency-based approach that favors rapid access to the least intensive intervention; some health care professionals in this study specifically suggested using a stepped care model in the future (Table 4). Stepped care can be used as a framework by which to deliver virtual care, by tailoring the intervention to the person’s needs, preferences, and readiness for behavioral changes in the context of chronic pain management.^37^ Interventions can span the continuum of care and include educational material, access to self-directed targeted treatment modules, peer support, group or individual treatment, and/or specialist care. This approach empowers patients to participate actively in care options, decisions, and delivery by allowing them to get the right care, at the right time, with a focus on virtual options.

### Strengths and Limitations

To our knowledge, no other group has sought to characterize the experiences of Canadian health care professionals working with youth having chronic pain and their families during the COVID-19 pandemic. This study engaged just over 150 health care professionals from a variety of settings with a wide variety of health care backgrounds, reflecting the multidisciplinary nature of pediatric pain care in Canada. Finally, this survey included several open-ended questions that allowed participants to share their unique concerns, suggestions for improvement, and potential solutions to some of the most pressing challenges that arose for front-line pain care professionals during the COVID-19 pandemic.

Limitations of this study include the generalizability of findings that were collected at a specific point in time; that is, during a rapidly changing pandemic context. Due to the rapid nature of this project and focus on feasibility, there may be additional questions that are important to answer such as whether there have been more new patients seeking care at tertiary care clinics or how many patients had to be added to a waiting list and therefore did not get any immediate treatment. This study sought to provide a high-level understanding on the current state of pediatric pain care delivery and practice, and we are conducting follow-up qualitative interviews in order to further understand some of the key concerns of HCPs. It is also not possible to know how representative the sample of health care provider respondents was among those invited to participate, because the survey was widely distributed among networks and we used a convenience sampling strategy. In addition, we were unable to survey all subspecialties where youth with pain may present (i.e., gastroenterology, adolescent medicine, physiatry, etc.) due to the rapid nature of the research and the barriers to obtaining permissions to access members and distribute surveys. Second, this study reflects the privileged perspective of medical directors and health care professionals but does not include the perspectives of patients and their families or data from administrative or health records. It is possible that some of the data reported in this survey were obtained through more objective measures (i.e., the number of requests for new patients or symptom report measures administered to patients); however, in order to provide a rapid report on the current practice, the study team chose to focus on aspects of the provision of pediatric chronic pain care that could be directly reported by medical directors and health care providers. Additionally, medical directors reported their perceptions of the patients who access tertiary-level clinics; this may thus exclude the experiences of underprivileged, non-white or Indigenous children or those who were unable to access care prior to the pandemic. A separate survey as well as interviews are currently being conducted to better understand this important patient perspective. Finally, a relatively small number of responses came from community or primary care settings (*n* = 15, 10%), and there were no respondents from Prince Edward Island or the three Canadian territories (Northwest Territories, Yukon, and Nunavut); notably, this province and the three territories do not have pediatric tertiary care hospitals and rely on their southern counterparts for specialized pain management.

## Conclusion and Future Directions

The formative data generated from this study revealed that 100% of pediatric chronic pain clinics across Canada rapidly implemented virtual care. This study also characterized the unique experiences of health care professionals working with the pediatric chronic pain population and highlighted some of the major challenges related to the provision of virtual care. A major concern is that even in a high-resourced country such as Canada, reliable Internet availability and access was the greatest challenge, which highlighted inequities in our health care system in rural or remote communities. Overall, this work highlighted the ability of pediatric chronic pain clinics to rapidly respond to the COVID-19 pandemic by shifting to delivering multidisciplinary pain management using virtual care solutions. Simultaneously, survey respondents highlighted the myriad of challenges that accompany this rapid shift to virtual care and identified potential strategies to consider moving forward in the development of virtual care guidelines and policies in Canada. These findings may be helpful in improving the availability of treatment for patients with chronic pain as a result of the implementation of virtual care solutions. Additionally, these results can inform the rapid design and implementation of tailored strategies, resources, and policy recommendations aligned with the work already underway through the Canadian Pain Task Force^2,31^ to meet identified needs for both health care professionals and youth living with chronic pain. The results of this study will be used to inform a consensus conference and consultation with decision makers to help develop consensus on virtual care priorities. In order to continue enhancing access to virtual pain care, the final phase of this project involves the development and usability testing of an online Kids Pain Portal, which will strengthen adaptive mental health and substance use care system capacity and flexibility and improve the availability of treatment for youth with chronic pain by leveraging virtual care solutions. By outlining the challenges and gaps in care, the results of this study provide useful data that will inform future guidelines and recommendations for virtual chronic pain care delivery in Canada.

## Supplementary Material

Supplemental Material

## Data Availability

The data that support the findings of this study are available from the corresponding author upon reasonable request.
