# Supplementary material for: COVID-19 Pandemic Impact and Response in Canadian Pediatric Chronic Pain Care: A National Survey of Medical Directors and Pain Professionals
Source: Can J Pain. 2021 Jun 30;5(1):139–50. doi: 10.1080/24740527.2021.1931069 (PMC8253119; doi:10.1080/24740527.2021.1931069)
Supplement: Supplemental Material [file UCJP_A_1931069_SM4259.zip › Supplementary material_Study 1 (clinic director) survey.docx]

Supplementary material: Survey 1 - HCP Clinic Director Survey

**Part 1:**

1. In what city or town is your pain clinic located?
2. Approximately how many patient visits does your clinic provide per year?
3. Has your clinic had to stop or significantly reduce in-person patient appointments? Yes/No
4. Are you providing care by other means? Yes/No
5. What means are you using? Phone/internet/other, provide detail _____
6. Are you trying to see all patients or are you triaging?
   1. All patients seen/Triage
   2. If applicable, what criteria are you using for triage?
7. Are you still able to see new patients or are they being added to wait list or a combination?
8. No new patients/some new patients/combination of seeing new patients and adding them to a waitlist
9. Are any procedures continuing? Yes/No
10. If yes, what procedures and what criteria are you using for urgent procedures?
11. What have been the biggest obstacles to providing virtual care?
12. How do you see this/these obstacle(s) being resolved?
13. In general, have your patients reported increases in pain? Yes/No
14. If yes, what are key reasons? [Select all that apply]
15. Decreased access to complementary treatments
16. Decreased access to other treatments
17. Decreased ability to exercise
18. Increased stress related to Covid-19 situation
19. Other ________
20. In general, has medication use increased? Yes/No
21. If so, what medication groups?
22. Antidepressants
23. Anticonvulsants
24. Opioids
25. Cannabinoids
26. Other (please name)________________
27. Have you had other issues with medications for patients? Yes/No
28. If yes, describe the issues
29. Are you still delivering physical, psychological and/or educational programs, either in person or virtually? Yes/No
30. If yes, what programs and how?
31. Are patients waiting longer for these programs? If yes, why?
32. What federal, provincial or territorial support would be helpful to facilitate providing virtual care?
33. What virtual practices do you anticipate your clinic will continue once the COVID crisis is over?
34. Do you have any other comments or lessons learned while providing care during the COVID-19 pandemic?

**Part 2**

Since March 2020 (when COVID-19 restrictions began):

1. Have you seen an increase or decrease in the number of requests to be seen from existing chronic pain patients? Increase/ No Change/ Decrease. Comments?
2. Have you had an increase or decrease in consults for new chronic pain patients?  Increase/ No Change/ Decrease. Comments?
3. Have you seen more patients with chronic pain having pain flares? Yes/ No. Comments?
4. Have you seen more pain flares leading to hospital admission? Yes/ No. Comments?
5. Are you seeing an increase in anxiety related symptoms? Yes/ No. Comments?
6. Are you seeing an increase in post-traumatic stress related symptoms? Increase/ No Change/ Decrease. Comments?
7. Are you seeing an increase in depressive symptoms? Increase/ No Change/ Decrease. Comments?
8. Are you seeing an increase in insomnia or sleep disturbance? Increase/ No Change/ Decrease. Comments?
9. Are you getting more requests for opioid or other analgesic prescriptions either from established or new patients? Yes/ No. Comments?
10. Are you seeing any increase in any substance misuse in the past few months? Yes/ No. Comments?
    1. If yes to Q10]: Which substance(s) [Check all that apply]: Cannabis/Alcohol/Opioids/Other (specify)
11. Does your clinic routinely screen youth with chronic pain for any mental health issues/disorders? Yes to everyone (specify)/ No/ Sometimes
    1. Which of the following does your clinic routinely screen for? Anxiety/Depression/Post-traumatic stress disorder/Insomnia or sleep disturbances/ Substance use disorders. Comments?
    2. [If yes]: What screening tool is most frequently used?
12. Does your clinic have a formal pathway for referring youth with CP to substance use disorder counselling? Yes (please describe)/ No
13. What are the barriers to your patients with chronic pain accessing services for mental health issues/disorder (e.g., depression, anxiety, PTSD, sleep disturbances)?
14. What are the barriers to your patients with chronic pain accessing services for substance use disorder?
