# Supplementary material for: COVID-19 Pandemic Impact and Response in Canadian Pediatric Chronic Pain Care: A National Survey of Medical Directors and Pain Professionals
Source: Can J Pain. 2021 Jun 30;5(1):139–50. doi: 10.1080/24740527.2021.1931069 (PMC8253119; doi:10.1080/24740527.2021.1931069)
Supplement: Supplemental Material [file UCJP_A_1931069_SM4259.zip › Supplementary material_Study 2 (individual HCP) Survey.docx]

Supplementary material: Study 2 Survey (Individual HCP)

**Part 1:**

1. *Has your emergency department seen changes in daily volume of patient presentations during the current pandemic? (if yes, describe)
2. **Have you or your clinic had to stop or significantly reduce in-person patient appointments? Yes/No/ Other (Please specify: ________)
3. Are you providing care by means other than in-person care? Yes/No
   1. What means are you currently using? [Select all that apply}: Telephone without video/ Internet or video calling / Other (please describe)
      1. [If internet or video calling]: Please specify which programs you use: Provincial system (e.g., Ontario Telemedicine Network [OTN]), Zoom, Other (Specify).
   2. If you are a healthcare provider that typically bills the provincial health care plan, have you been able to bill for these visits? Yes/ No/ Not applicable/ Other (describe).
   3. What have been the biggest obstacles to providing virtual care? Technical barriers (if yes, specify hardware and/or software barriers, and is the technical issue on your end and/or on the end of the patient?) / Financial barriers / Issues related to medications (e.g., difficulty getting prescriptions to pharmacists, other?) If yes, please describe) / Other (describe)
   4. How do you see this/these obstacle(s) being resolved?
4. **Are you still delivering physical, psychological and/or educational programs, either in person or virtually? Yes/No/Other (Please specify)
   1. If yes, what programs and how?
   2. Are patients waiting longer for these programs? If yes, why?
5. **Have you noticed a change in the rate of attendance of patients for their appointments since implementing virtual care? Yes – improved attendance / Yes – worse attendance / No Change / Not applicable
6. What federal, provincial or territorial support would be helpful to facilitate providing virtual care?
7. What virtual practices do you anticipate your clinic will continue once the physical distancing measures have been lifted?
8. Do you have any other comments or lessons learned while providing care during the COVID-19 pandemic, in the context of connecting with patients?

* Question only included for emergency department clinicians

** Question not included for emergency department clinicians
